# The olfactory nerve is not a likely route to brain infection in COVID-19: a critical review of data from humans and animal models

**DOI:** 10.1007/s00401-021-02314-2

**Published:** 2021-04-26

**Authors:** Rafal Butowt, Nicolas Meunier, Bertrand Bryche, Christopher S. von Bartheld

**Affiliations:** 1 L. Rydygier Collegium Medicum, Nicolaus Copernicus University, 85-094 Bydgoszcz, Poland; 2 Université Paris-Saclay, INRAE, UVSQ, VIM, 78350 Jouy-en-Josas, France; 3 Department of Physiology and Cell Biology, School of Medicine, University of Nevada, Reno, Reno, NV 89557 USA

**Keywords:** SARS-CoV-2, COVID-19, Olfactory system, Neuro-invasion, Brain infection, Virus

## Abstract

One of the most frequent symptoms of COVID-19 is the loss of smell and taste. Based on the lack of expression of the virus entry proteins in olfactory receptor neurons, it was originally assumed that the new coronavirus (severe acute respiratory syndrome coronavirus 2, SARS-CoV-2) does not infect olfactory neurons. Recent studies have reported otherwise, opening the possibility that the virus can directly infect the brain by traveling along the olfactory nerve. Multiple animal models have been employed to assess mechanisms and routes of brain infection of SARS-CoV-2, often with conflicting results. We here review the current evidence for an olfactory route to brain infection and conclude that the case for infection of olfactory neurons is weak, based on animal and human studies. Consistent brain infection after SARS-CoV-2 inoculation in mouse models is only seen when the virus entry proteins are expressed abnormally, and the timeline and progression of rare neuro-invasion in these and in other animal models points to alternative routes to the brain, other than along the olfactory projections. COVID-19 patients can be assured that loss of smell does not necessarily mean that the SARS-CoV-2 virus has gained access to and has infected their brains.

## Introduction

It is now well established that nearly half of all patients with COVID-19 have a reduction or loss of smell as one of their symptoms [97], resulting in tens of millions of cases of—for the most part transiently—reduced smell. Since some viruses can be “neuro-invasive,” meaning that they can enter the nervous system, there has been a concern that the new coronavirus, SARS-CoV-2, may reach the brain, using the nose as a portal, as is known or suspected for a subset of other viruses [33, 73, 95]. Is there convincing evidence that SARS-CoV-2 can infect olfactory neurons and can travel along their axons from the nose to the brain? It is known that—in rare cases—SARS-CoV-2 is present in the human brain [34, 65, 66, 68, 75, 86, 88], and it was suggested that infection of respiratory centers in the brainstem may contribute to fatal outcomes in COVID-19 [5, 31, 40, 54, 58, 63, 64, 89]. Since a large number of patients with COVID-19 lose their sense of smell, do all these people have to live in fear about a subsequent brain infection, as recent provocative titles of publications would suggest: “Olfactory transmucosal SARS-CoV-2 invasion as a port of central nervous system entry in individuals with COVID-19” [66] and “SARS-CoV-2 invades the central nervous system via the olfactory route in Rhesus monkeys” [51]? In this review, we critically evaluate the current evidence whether SARS-CoV-2 may utilize olfactory neurons as a route to brain infection.

## Can olfactory neurons become infected by SARS-CoV-2?

Because of the high viral load in the nasal epithelium [48, 66, 83, 100, 107] and because of the proximity of the nasal cavity to the skull and brain, many investigators have considered the possibility that SARS-CoV-2 travels from the nose to the brain along the olfactory nerve [10, 14–16, 18, 19, 59, 66, 72, 81, 108], similar to some of the other neuro-invasive viruses [29, 50, 89]. The cellular and molecular consequences of SARS-CoV-2 infection in the olfactory epithelium have now been examined in increasing detail, in multiple animal models as well as in human cell and tissue samples obtained by brush sampling, through biopsy, and post-mortem analysis (Tables 1, 2 and 3).

**Table 1:** Mouse studies reporting ACE2 expression or SARS-CoV-2 infection in the olfactory epithelium, virus presence in the brain, and loss of smell

Authors	Date of publication (preprint) 2020/2021	ACE2 expression models	ACE2 in identified cell types		SARS-CoV-2 in identified cell types		dpi examined (brain)	Virus in brain?	Method of virus detection in the brain	Behavior: loss of smell
			SC	ORN	SC	ORN				
Bilinska et al. [8]	5/7/20	WT mouse	Most	No						
Baxter et al. [6]	(5/15/20)	WT mouse		No						
Ziegler et al. [106]	5/28/20	WT mouse	Some							
Brann et al. [11]	(5/18/20) 7/28/20	WT mouse	Yes	No						
Leist et al. [57]	9/12/20	WT mouse, mouse-adapted virus			Yes		2	No	PCR	
Bao et al. [2]	5/7/20	hACE2 mouse: exogenous murine ACE2 pr					1, 3, 5, 7	No	PCR	
Hassan et al. * [45]	8/16/20	hACE2 mouse: AV, CMV pr					4	"Low”	PCR	
Rathnasinghe et al. * [80]	11/6/20	hACE2 mouse: AV, CMV pr					2, 5	No	PF, ICn	
Sun et al. [91]	6/10/20	hACE2 mouse: murine ACE2 pr					6	Yes, at 6 dpi	PCR, ICs	
Zhou et al. [105]	(10/27/20)	hACE2 mouse: endogenous murine ACE2 pr					2, 4	Yes, some at 4 dpi	PCR	
Ye et al. [98]	(11/10/20)	hACE2 mouse: endogenous murine ACE2 pr	Most	Very few	Most	Yes at 4 dpi	2, 4	No, except PCR, at 2 dpi	PCR, ISH, ICn	Yes, at 2 dpi
Jiang et al. [51]	(5/21/20) 7/9/20	hACE2 mouse: HFH4/FOXJ1 pr					1, 3, 5, 7	Yes, at 5–7 dpi	PCR	
Yinda et al. [99]	(8/11/20) 1/19/21	hACE2 mouse: K18 pr					3, 7	Yes, at 3, 7 dpi (IC)	PCR, ICn	
Winkler et al. [101]	(8/24/20) 2/24/21	hACE2 mouse: K18 pr					2, 4, 7	Yes, at 2, 4, 7 dpi	PCR, PF	
Song et al. [89]	(9/8/20) 1/12/21)	hACE2 Mouse: K18 pr					2, 4, 7	Yes, at 2, 4, 7 dpi	PCR, ICn, PF	
Golden et al. [41]	10/2/20	hACE2 mouse: K18 pr					3, 5–11	Yes, at 5–11 dpi	ISH, ICs	
Rathnasinghe et al. [80]	11/6/20	hACE2 mouse: K18 pr					2, 5, 6	Yes, at 2, 5, 6 dpi	PF, ICn	
Zheng et al. [104]	(8/10/20) 11/9/20	hACE2 mouse: K18 pr			Yes		2, 4, 6	Yes, at 4, 6 dpi	PF, ICn	Yes, at 2–3 dpi
Oladunni et al. [76]	11/30/20	hACE2 Mouse: K18 pr					2, 4, 6	Yes, at 4, 6 dpi	PF	
Kumari et al. [54]	(12/14/20)	hACE2 mouse: K18 pr					1, 3, 5, 6	Yes, at 3–6 dpi	PCR, PF, ICs	
Carossino et al. [21]	(1/13/21) 1/19/21	hACE2 mouse: K18 pr					2, 4, 6/7, 14	Yes, at 4, 6/7 dpi	ICs, ISH	

*ACE2* angiotensin-converting enzyme 2, *AV* adenovirus vector, *CMV* cytomegalovirus, *dpi* day post infection, *hACE2* human angiotensin-converting enzyme 2, *HFH4/FOXJ1* human forkhead box J1 (FOXJ1) promoter, *ICn* immunocytochemistry against the nucleocapsid protein, *ICs* immunocytochemistry against the spike protein, *ISH* in situ hybridization, *K18* cytokeratin K18 promoter, *olf*. olfactory, *ORN* olfactory receptor neuron, *PCR* polymerase chain reaction, *PF* plaque formation, *pr* promoter, *SARS-CoV-2* severe acute respiratory syndrome coronavirus 2, *SC* sustentacular cell, *WT* wild-type

*These mice use CMV as promoter for ACE2 expression with yet uncharacterized expression in different cell types

**Table 2:** Hamster and ferret studies reporting ACE2 expression or SARS-CoV-2 infection in the olfactory epithelium, virus presence in the brain, and loss of smell

Authors	Date of publication (preprint)	Species	ACE2 in identified cell types		SARS-CoV-2 in identified cell types		dpi examined (brain)	Virus in brain?	Method of virus detection in the brain	Behavior: loss of smell
	2020/2021	ACE2 expression	SC	ORN	SC	ORN				
HAMSTER										
Chan et al. [22]	3/26/20	WT Hamster					2, 4, 7	No	PCR	
Sia et al. [87]	5/14/20	WT Hamster				Maybe	2, 5, 7	No?	ICn	
Bryche et al. [15]	7/3/20	WT Hamster			Yes	No	2, 4, 7, 10, 14	No	ICn	
Imai et al. [49]	7/14/20	WT Hamster					3, 6, 10	Yes, at 3 dpi	PF	
Zhang et al. [103]	7/15/20	WT Hamster	Most	Some	Most	Few	0.5, 2, 4, 7, 14	No	ICn	
de Melo et al. [28]	(11/18/20)	WT Hamster			Yes	Few	2, 4, 14	No, except PCR at 2, 4 dpi	PCR, ICn	Yes, at 3, 5 dpi
Hoagland et al. [46]	1/29/21	WT Hamster					1, 2, 4, 6, 8, 14 dpi	Yes, with PCR, 1 case con-firmed by PF	PCR, PF	
Zazhytska et al. [102]	(2/9/21)	WT Hamster			Yes	No				
FERRET										
Schlottau et al. [84]	7/7/20	WT Ferret					4, 8, 12, 21	No, except PCR, at 8, 21 dpi	ICn, ISH, PCR	
Everett et al. [35]	1/15/21	WT Ferret			Yes	Maybe	3, 5, 7	No, except PCR	ICn, ICs, ISH, PCR	

*ACE2* angiotensin-converting enzyme 2, *dpi* day post infection, *ICn* immunocytochemistry against the nucleocapsid protein, *ICs* immunocytochemistry against the spike protein, *ISH* in situ hybridization, *olf*. olfactory, *ORN* olfactory receptor neuron, *PCR* polymerase chain reaction, *PF* plaque formation, *SARS-CoV-2* severe acute respiratory syndrome coronavirus 2, *SC* sustentacular cell, *WT* wild-type

**Table 3:** Human and non-human primate studies reporting ACE2 and TMPRSS2 expression or SARS-CoV-2 infection in the olfactory epithelium or virus presence in the brain

Authors	Species	Date (preprint)	ACE2 in cell types		TMPRSS2 in cell types		SARS-Cov2 in cell types		Virus in brain?	Method of detection in brain
		2020/2021	SC	ORN	SC	ORN	SC	ORN		
MACAQUE										
Munster et al. [70]		5/12/20							None, at 3, 21 dpi	PCR
Rockx et al. [83]		5/29/20							None, at 4, 21 dpi	PCR
Deng et al. [27]		9/2/20							Minimal at 7 dpi	PCR
Jiao et al. [51]		(10/20/20)							Yes, at 1, 4, 7 dpi	PCR, ICn
Philippens et al. [78]		(2/23/21)							Yes, at 35 dpi by PCR	PCR, ICn
CERCOPITHECUS										
Hartman et al. [44]		9/18/20							Yes, at 28, 35 dpi	PCR
HUMAN										
Chen et al. [23]		(5/9/20) 9/24/20	Yes	No						
Nampoothiri et al. [71]		(6/19/20)							Yes, in choroid plexus, olf. bulb, hypothalamus	ICn, ICs
Ellul et al. [34]		7/2/20							Rarely positive in CSF	PCR
Klingenstein et al. [52]		(7/15/20) 1/22/21	Most	No	Most	No				
Brann et al. [11]		(5/18/20) 7/28/20	Yes	No	Yes	No				
Gupta et al. [42]		(4/1/20) 8/18/20	Some	No						
Song et al. [89]		9/8/20							Yes, in cortex, and subcortical vessels	ICs
Matschke et al. [65]		10/5/20							Yes, in forebrain, brainstem	PCR, ICn, ICs
de Melo et al. [28]		(11/18/20)					Yes	Yes		
Cantuti-Castelvetri et al. [20]		(6/10/20) 11/13/20	Yes	?			Yes	?		
Neumann et al. [74]		11/15/20							No virus in CSF	PCR
Fodoulian et al. [38]		(4/2/20) 11/25/20	Yes	No	Yes	No				
Meinhardt et al. [66]		(6/4/20) 11/30/20	Yes	No				?	Yes, various sites medulla > olf. bulb	PCR, ICs
Nuovo et al. [75]		12/24/20							Very rare with ISH, ICs: in vessels, microglia	ISH, ICs
Mukerji and Solomon [68]		1/18/21							Yes, with PCR, rare with ICn, ICs	PCR, ICn, ICs
Zazhytska et al. [102]		(2/9/21)					Yes	No		

*ACE2* angiotensin converting enzyme 2, *CSF* cerebrospinal fluid, *dpi* day post infection, *ICn* immunocytochemistry against the nucleocapsid protein, *ICs* immunocytochemistry against the spike protein, *PCR* polymerase chain reaction, *olf.* olfactory, *ORN* olfactory receptor neuron, *SARS-CoV-2* severe acute respiratory syndrome coronavirus 2, *SC* sustentacular cell, *TMPRSS2* Transmembrane protease serine 2, *?* questionable

To assess whether olfactory receptor neurons may be susceptible to infection by SARS-CoV-2, investigators have determined which cell types in the olfactory epithelium express the obligatory entry proteins for the new coronavirus, angiotensin-converting enzyme 2 (ACE2) and transmembrane protease, serine 2 (TMPRSS2). These gene and protein expression studies were performed by RNAseq of identified cell types, or using markers for distinct cell types within the olfactory epithelium combined with gene or protein expression for ACE2 and TMPRSS2 [6, 8, 11, 23, 38, 42, 52, 66, 98, 103, 106]. The large majority of these studies concluded that sustentacular cells (the primary support cells in the olfactory epithelium) and cells in Bowman’s glands express the virus entry proteins, while all human studies and the majority of animal studies reported that olfactory receptor neurons do not express ACE2, or express ACE2 only very rarely (Tables 1, 2 and 3).

Another series of studies examined where in the olfactory epithelium the new coronavirus accumulates, by employing in situ hybridization or antibodies against viral proteins in histological sections [15, 20, 28, 66, 87, 98, 102, 103] or by brush sampling [28]. Some of these studies conducted double-labeling with antibodies against viral proteins as well as antibodies for specific cell types in the olfactory epithelium [15, 20, 28, 66, 87, 98, 103]. While the data of most of these studies show that the sustentacular cells are the main type of cells accumulating the virus, consistent with the predictions of the virus entry protein studies, some investigators reported that the virus can also be found in olfactory receptor neurons [28, 35, 66, 87, 98, 103]. Whether olfactory neurons become infected is an important question because of the possibility of axonal transport of the virus from the nose into the brain. Neuro-invasive viruses that use the olfactory route are known to bind with high affinity to olfactory receptor neurons [33, 56, 95].

## Explanations for contradictory reports on neuron infection and neuro-invasion

How can the contradictory findings between studies on the expression of entry virus proteins and several of the virus-localization studies be reconciled? Does the virus indeed accumulate in olfactory receptor neurons (and their axons), and not only in the sustentacular cells and gland cells? Important peculiarities of the olfactory system may explain why different studies have arrived at different conclusions.

**Fig. 1:**
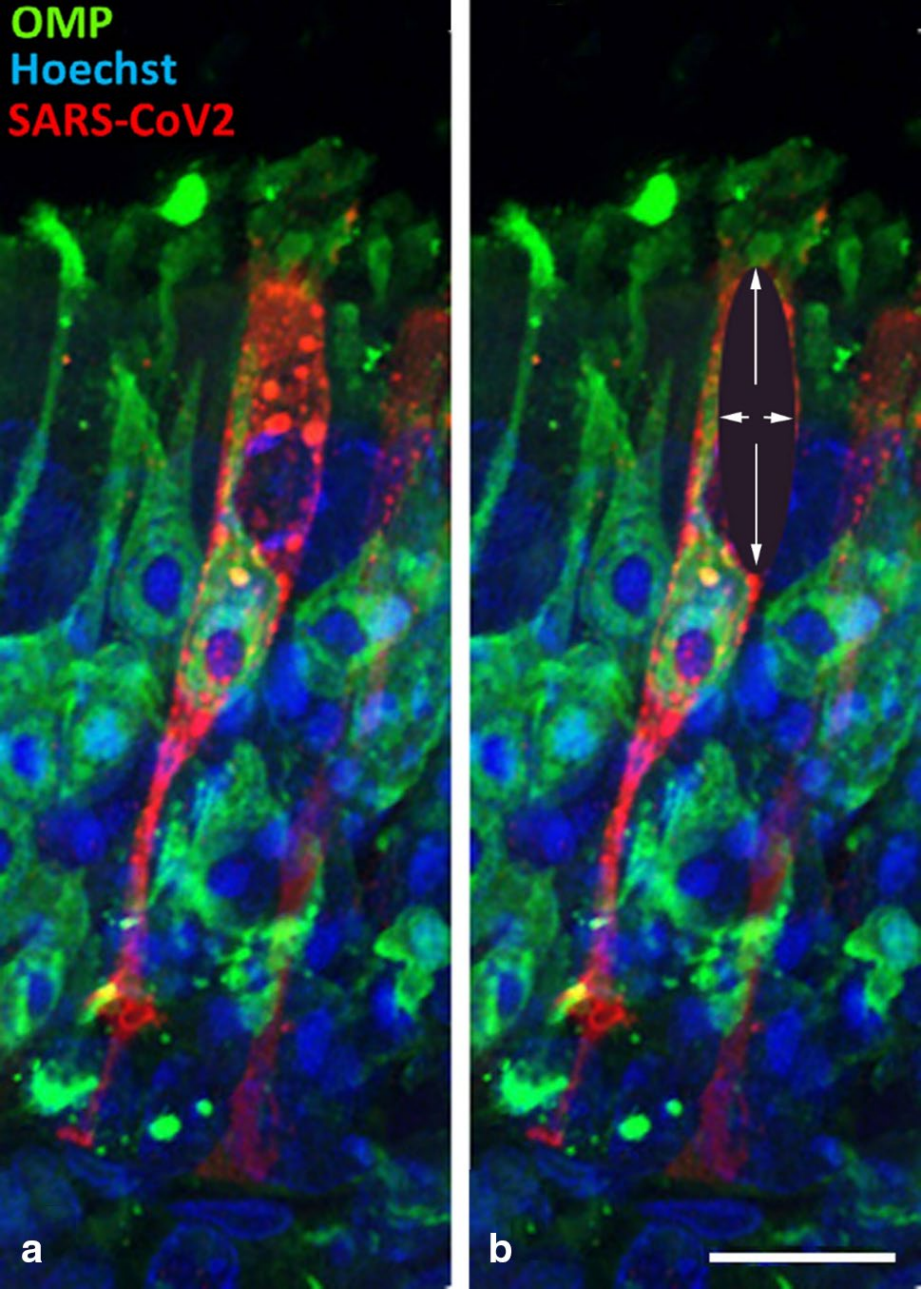
SARS-CoV-2 nucleocapsid protein (red) immunolabeled in the olfactory epithelium, double-labeled for olfactory marker protein (OMP, green) and stained with Hoechst nuclear stain (blue). **a** The SARS-CoV-2 (red) is present in a sustentacular cell that partially overlaps with an OMP-labeled olfactory receptor neuron. **b** When in the same image the sustentacular cell body is invisible (black ellipsoid shadow with white arrows), as it would be when the plane of section is not entirely perpendicular to the epithelium, then the SARS-CoV-2 protein would be erroneously interpreted to be co-localized within the OMP-expressing olfactory receptor neuron. Scale bar = 10 µm. Image is adapted from Bryche et al. [15]

1. The olfactory epithelium contains millions of olfactory receptor neurons and sustentacular cells. Most of the studies reporting an infection of olfactory neurons by SARS-CoV-2 do not provide a quantitative analysis. They describe few examples of putative olfactory neurons containing SARS-CoV-2 and display high magnification images of the olfactory epithelium showing isolated olfactory neurons possibly co-labeled for SARS-CoV-2. Studies that examined SARS-CoV-2 distribution semi-quantitatively showed that the virus mostly localizes to sustentacular cells and Bowman’s gland cells [15, 57, 98], while olfactory neurons do not contain SARS-CoV-2 or contain it only rarely [15, 98, 103, 104]. The only proper quantification so far was made in human ACE2 transgenic mice [98], and the authors found only 0.03% of mature olfactory receptor neurons to be infected.
2. Virus-infected olfactory epithelium has been shown to contain dying neurons. Some of these dying neurons are phagocytosed by immune cells [28], but they can also be phagocytosed by sustentacular cells [85, 92], ensuring the removal of receptor neurons that die due to constant turnover [53]. Accordingly, some sustentacular cells will contain phagocytosed proteins that are normally found only in olfactory neurons, possibly including neuronal markers. This can create false positives, because the viral protein of an infected sustentacular cell that phagocytosed a neuron or neuronal debris may appear as an example of a cell containing viral protein co-localized with a neuronal marker protein, even when the dying neuron downregulates such marker proteins. Indeed, virus-infected olfactory epithelium has been shown to contain dying neurons (recognized by their fragmented nuclei and chromatin condensation), some of which were being phagocytosed [28]. Accordingly, occasional co-localization of neuronal and viral proteins may generate false positives.
3. Sustentacular support cells tightly wrap olfactory receptor neurons, and especially their dendrites extending towards the nasal cavity [13, 60, 61]. This makes it difficult to distinguish between protein content within the neuronal compartment and protein content within the support cell compartment [98]. Accordingly, at a superficial glance and by merging confocal images at the light-microscopic level, the two labels may appear to overlap, when they actually are in distinct spaces, just very close together. This is illustrated in Fig. 1 adapted from the work of Bryche et al. [15] where it can be seen that only one cell, a sustentacular cell, contains the virus (Fig. 1a, red label), and not the adjacent neuron, but this is apparent only when the top of the labeled sustentacular cell is visible within the same tissue section (Fig. 1a)—if the section had been cut too thin or anything less than perfectly perpendicular to the plane of the epithelium, the virus would have been deemed to be located within the neuron (Fig. 1b). The entwinement of olfactory neurons with their support cell may explain why some studies reported viral protein in olfactory neurons, when the viral protein in fact may have been present in the tight wrappings of sustentacular cells [28, 35, 66, 87, 98, 103].
4. Examination at the electron microscopic level presents an alternative approach to avoid the false positive evaluation that may arise from fluorescent-based localization of cell type-specific markers. Only two studies have explored the cellular localization of the virus in the olfactory epithelium with this technique [28, 66]. However, both studies may have misinterpreted their images. Ciliated respiratory cells differ from ciliated dendritic knobs of olfactory receptor neurons at the ultrastructural level (see, *e.g.*, [36], their Fig. 3a, c). Fig. 3c–f in Meinhardt et al. [66] shows virus protein in such a ciliated cell, not in an olfactory neuron’s ciliated dendritic knob. Furthermore, the arrows in Fig. 3a in [66] may indicate the luminal portions of sustentacular cells, not a dendritic knob, because the size of one of these “knobs” is incompatible with the known size of knobs (they are about 1–3 µm in diameter [37, 52]), as we and others have previously pointed out [7, 19, 24]. Accordingly, current evidence for SARS-CoV-2 or viral protein in the dendrites of olfactory receptor neurons is questionable. Similarly, some error seems to have been made in the interpretation of respiratory and olfactory epithelium [28]. Their Fig. 4b and e display respiratory ciliated cells which are interpreted as belonging to the olfactory epithelium.
5. In the few studies that describe SARS-CoV-2 localized in olfactory receptor neurons, the virus appears to be more often localized in immature rather than mature olfactory receptor neurons, consistent with the lack of ACE2 expression in mature olfactory neurons [5, 24] (Tables 1, 2 and 3). However, as explained in detail below, the immature neurons do not yet have axons that extend to their target glomerulus in the olfactory bulb [53, 61, 62, 85]. Therefore, when their cell bodies were infected in the olfactory epithelium, they would not be able to carry the virus to the olfactory bulb. Even if SARS-CoV-2 persists in such neurons until they are mature, the time required for maturation far exceeds the time at which the virus arrives in the brain in animal models.
6. It is interesting that most of the virus-containing axons shown by de Melo et al. [28] in the olfactory nerve (their Fig. 5e) do not express olfactory marker protein (OMP), and therefore either are axons of olfactory neurons that have ceased to express OMP, (possibly because they are dying due to the infection [67, 103]), or these axons are not olfactory axons. It is rarely appreciated that some axons in the olfactory nerve are not derived from first-order olfactory neurons that project to the glomeruli in the olfactory bulb but are axons of nervus terminalis neurons that bypass the glomeruli in the olfactory bulb and project to various targets in the forebrain [29, 55, 96], and many of these neurons express ACE2 [9]. The nervus terminalis is a heterogeneous complex of nerve fibers and ganglia that connects the olfactory epithelium with targets in the forebrain caudal to the olfactory bulb [29].
7. Some of the studies localizing SARS-CoV-2 in the brain used antibodies against the spike protein to document virus localization [21, 41, 54, 66, 91]. However, it is now known that the S1 subunit of the spike protein can be shed from the virus during cell entry, and neurons in the brain can take up such cleaved and systemically circulating spike proteins [75, 82]. Accordingly, cells in the brain may contain spike proteins without necessarily containing SARS-CoV-2 virus. When localization of virus RNA was directly compared with localization of spike protein in human autopsy tissues, the large majority of the blood vessels in the brain containing spike protein did not contain any viral RNA [75].
8. Proponents of an olfactory route of SARS-CoV-2 to achieve brain infection often allude to “neuron-hopping” as the mechanism for travel into and within the brain [10, 14, 16, 28, 54, 63, 66, 72, 108]: virus transfer from olfactory receptor neurons to mitral cells (2^nd^ order neurons) in the olfactory bulb, and then transfer to 3^rd^ order neuronal targets in the brain. The time course of virus internalization and subsequent axonal transport by neurons is well established—it takes approximately 24 h for each virus transfer from one neuron to the next-order neuron [4, 33, 73], presumably due to the velocity of kinesin-mediated axonal transport [93]. Previous work established also that neuro-invasive viruses typically infect only structures neuroanatomically linked to the site of inoculation [90]. However, the time course of SARS-CoV-1 and SARS-CoV-2 invasion from the olfactory epithelium to distant targets in the brain, even those that are not 2nd or 3rd order olfactory targets, is much more rapid: the arrival of the virus is approximately simultaneous in the olfactory bulb and in distant brain targets [28, 73, 104], or even “skips” the olfactory bulb [21, 101, 105] or the glomerular layer containing the olfactory axons [104]. These findings do not support the hypothesis that SARS-CoV-2 invades the brain by multiple transfers from neuron to neuron, with the first transfer from olfactory receptor neurons to mitral cells in the olfactory bulb. The observed time course is more consistent with alternative routes of neuro-invasion [5, 39, 81]. Such alternative routes include a pathway that reaches cerebrospinal fluid (CSF)-containing spaces, uses the vasculature, or the virus may travel along a peripheral nerve such as the nervus terminalis that directly innervates the forebrain, including the hypothalamus [96].

Taken together, there are multiple explanations for the seemingly contradictory findings of whether or not olfactory receptor neurons can be infected by SARS-CoV-2 and can carry the virus into the brain. Virus localization within olfactory receptor neurons is ambiguous at best, and there is currently no convincing evidence that the virus travels from the nose to the brain along the axons of olfactory receptor neurons.

## When and how does the virus reach the brain?

Brain infection by SARS-CoV-2 has been studied and verified in animal models, primarily in mouse and hamster, in addition to more limited data on humans and non-human primates. Due to species differences of the ACE2 protein [25], SARS-CoV-2 infectivity varies between species. Wild-type mice have low infectivity for SARS-CoV-2, and to study infectivity and virus spread in this species, the SARS-CoV-2 virus has to be mouse-adapted [57, 69], or mice have to be engineered to express human ACE2 instead of, or in addition to, murine ACE2 (Table 1). Multiple such mouse models with different promoters have been developed (reviewed in [19, 43, 69, 76, 80]). Hamsters express an ACE2 protein that results in medium-to-high infectivity of SARS-CoV-2, more similar to the infectivity in humans, and hamsters are therefore deemed to be a more physiological animal model to study neuro-invasion in COVID-19 [24, 25, 28, 103]. In this context, it is important to understand the advantages and limitations of the methods that have been used to provide evidence for the presence of SARS-CoV-2 in tissues, as summarized in Table 4. Plaque formation provides evidence of replicating virus but does not inform about cellular localization. PCR gives evidence of viral RNA but does not inform about virus replication or cellular localization, and it is still uncertain whether subgenomic RNA is indeed an indicator of active replication [1]. In situ hybridization gives evidence of viral RNA and tissue localization. Antibodies against either the spike protein or the nucleocapsid of the virus provide cellular localization but do not distinguish whole virus from cleaved proteins that can circulate systemically in the brain [75]. Evidence at the ultrastructural level is rarely attempted and fraught with uncertainty [47].

**Table 4:** Advantages and limitations of the methods that have been used to prove SARS-CoV-2 in brain tissues

Method	What is detected?	Advantages	Limitations
Plaque formation (PF)	Replicating virus	Evidence for replication of virus	No cellular localization
RT-qPCR (PCR)	Virus mRNA	Highly sensitive for virus mRNA	Contamination may give false positives, no cellular localization, significance of subgenomic vs. genomic RNA for replication still uncertain
In situ hybridization (ISH)	Virus mRNA	Sensitive for virus mRNA	
Immunocytochemistry	Virus protein (antigen)	Cellular localization	
For nucleocapsid protein (ICn)	Nucleocapsid protein	Cellular localization	No distinction between shed protein and entire virus
For spike protein (ICs)	Spike protein	Cellular localization	No distinction between shed protein and entire virus
Electron microscopy	Virus	Precise tissue localization	Very difficult identification of virus even with good morphology

**Fig. 2:**
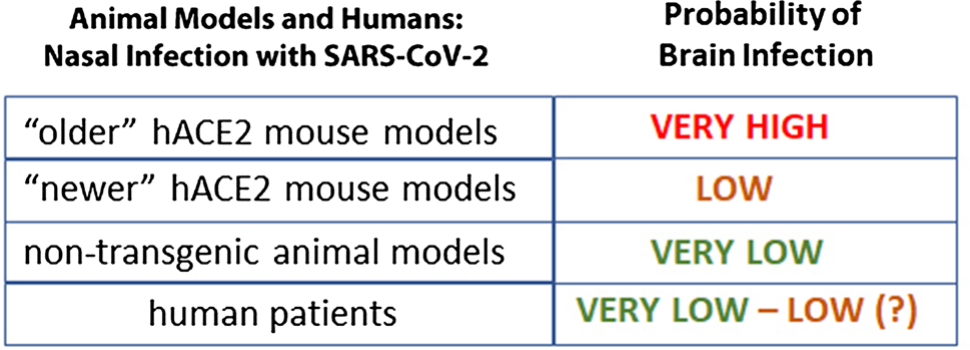
Probability of brain infection after nasal inoculation in animal models or in SARS-CoV-2 infected patients. Note that the probability of brain infection in humans resembles that in non-transgenic animal models and in the newer human ACE2 (hACE2) mouse models, but not the infection probability in the older transgenic mouse models that use the K18 cytokeratin promoter

In mice that express human ACE2 (hACE2), SARS-CoV-2 infects the olfactory epithelium. Brain infection after intranasal infection seems to depend on the type of promoter used to control the expression of hACE2, as compiled in Table 1 and summarized in Fig. 2. Mouse models that express hACE2 under the control of the endogenous or exogenous murine ACE2 promoter or the cytomegalovirus (CMV) promoter showed mild disease symptoms and only occasionally had SARS-CoV-2 in the brain [2, 45, 80, 89, 98, 105]. In these animals, evidence for virus presence was based mostly on PCR [91, 105], but was not detected by immunocytochemistry or in situ hybridization (Table 1). These “newer” mouse models were generated by CRISPR/cas9 and knock-in approaches, thus the endogenous ACE2 expression is replaced by human ACE2 expression; such mouse models are considered to be more physiologically relevant than the “older” mouse models, although the sometimes-used adenoviral vector may by itself elicit host responses separate from responses to the SARS-CoV-2 infection [43]. The “older” mouse models were generated several years ago for SARS-CoV-1 studies; they have constitutive hACE2 expression controlled by exogenous promoters such as K18 cytokeratin or forkhead box protein J1 (FOXJ1). These mouse models are more often lethal, presumably due to brain infection [21, 41, 50, 54, 76, 80, 89, 101, 104], while the “newer” mouse models and mice infected with mouse-adapted SARS-CoV-2 typically do not show neuro-invasion [57] (Table 1). Other viruses, such as human coronavirus OC43 (HCoV-OC43), mouse hepatitis virus, or herpes simplex virus, readily infect olfactory neurons and move effectively by anterograde axonal transport to secondary and tertiary olfactory centers in the brain [3, 4, 17, 33, 56, 95]. The reason why SARS-CoV-2 rarely infects olfactory circuits in the newer mouse models or in wild-type animals appears to be the lack of expression of the virus entry proteins in olfactory neurons. The viruses that are highly effective neuro-invaders have in common that their entry proteins are abundantly expressed in the neurons that become infected [3, 17, 33, 56, 95].

In some of the mouse models expressing human ACE2, the timing of arrival of the virus in various brain structures was monitored, and it was described that olfactory bulbs were infected not more than other parts of the brain, especially the hypothalamus and other thalamic nuclei, and the brainstem [21, 101, 104]—similar to some of the human neuropathological findings [66, 86], as described below. The need to engineer human ACE2 expression is a limitation of the mouse animal model, because hACE2 may not be expressed in the same cell types and at the same levels as it is expressed in humans; this may enable neuro-invasion of SARS-CoV-2 that would not occur with normal expression of ACE2. Data from such mice, therefore, need to be interpreted with caution [24]. On the other hand, transgenic mouse models inform about which types of neuro-invasion by SARS-CoV-2 are possible when certain cell types express certain levels of virus entry proteins, and these mice are an important animal model to test mechanisms of neuro-invasion as well as antiviral strategies [21, 104].

In hamsters, seven studies explored if the virus was present in the brain following SARS-CoV-2 nasal inoculation [15, 22, 28, 49, 87, 103]. Four of them found no evidence for brain infection with antibodies (Table 2). Three studies found either viral RNA or virus (by plaque formation) in the olfactory bulb or brain at 1 to 14 days post infection (dpi) [28, 46, 49], with 2 logs lower than in nasal turbinates and with similar levels in the brainstem, cerebral cortex and cerebellum [28]. In cases with positive PCR, virus presence could rarely be confirmed by plaque formation [46], indicating that the large majority of viral RNA was not replicating virus. Using immunohistochemistry or in situ hybridization, five studies reported the absence of viral antigens in the brain or olfactory nerve [15, 49, 87, 102, 103]. One study observed only a few infected (non-neuronal) cells in the olfactory bulb [28]. These discrepancies between studies could be related to the viral titer used during infection. Indeed, the virus titer varies up to 10,000-fold between studies, from 10 plaque-forming units (pfu) [46] to 1 × 10^5^ pfu [103], and the virus was rarely observed in the brain when lower virus titers were used for infection [77]. Similar observations were made in monkeys as described below.

In ferrets, ACE2 has a low virus-binding score, but these animals are susceptible to SARS-CoV-2 infection [25, 35, 84]. As shown in Table 2, SARS-CoV-2 localizes to presumptive sustentacular cells in ferrets [35], similar to hamsters. Only very few animals had virus in the brain clearly above the threshold of detection by PCR [84], and neuro-invasion could not be verified by other methods.

In the physiological animal models (hamster, ferret), the virus was found in the brain only by quantitative PCR or plaque formation, but not by immunocytochemistry (Table 2). This raises the question of the cellular source of the virus. Indeed, if the virus is present only in blood vessels or in circulating immune cells in the brain, virus presence may not be related to neuronal infection. Overall, the studies in hamsters and ferrets do not support brain infection by an olfactory route.

In non-human primates (macaques and African green monkeys), six studies examined SARS-CoV-2 in the brain after nasal or upper respiratory tract inoculation. The first three studies did not find evidence of the virus in the brain using PCR at 3, 4, 7, and 21 days after infection [27, 70, 83] (Table 3). A fourth and fifth study [44, 78] found viral RNA in multiple brain regions at 28 or 35 days post infection, but in one of these studies, the PCR findings could not be verified by antibodies against the nuclear capsid antigen [78]. A sixth study [51] found evidence of virus RNA and nuclear capsid antigen in the brain, including the olfactory bulb, at 1, 4 and 7 days after nasal inoculation. However, this study applied an extremely high dose of virus (10^7^ pfu), about 20 times higher than the other monkey studies (0.7 × 10^5^ and 3 × 10^5^), and more than 100 times the dose of most other animal studies (5 × 10^3^ to 10^5^ pfu). Jiao et al. [51] found viral RNA in the blood and in the CSF already at day 1 after nasal infection. Such a fast appearance of the virus in the CSF essentially precludes neuronal transfer along the olfactory nerve as the sole or primary pathway and instead points to alternative routes of SARS-CoV-2 to achieve brain infection.

In humans, there are no time course studies of neuro-invasion, only reports on the “final outcome.” The virus was found to be abundant in the olfactory epithelium, mostly, if not exclusively, in sustentacular cells [20, 28, 66]. In some patients, the virus was also documented in the brain, with the brainstem, thalamus and hypothalamus more often infected than the olfactory bulb [66]. Virus was also documented in some cases in the cerebral cortex and in the CSF or choroid plexus [16, 26, 34, 63, 65, 66, 71, 75, 86, 89], but it was not detectable in the CSF in other studies [74, 79].

Could the small number of potentially infected olfactory receptor neurons contribute to neuro-invasion of the brain in animals and humans? Most of the reported examples are immature neurons. Immature olfactory receptor neurons cannot transmit the virus to the brain, because they do not have the peripheral and central connections: after the 7–14 days required for the generation of neurons [53], it takes another several day for the immature olfactory neurons to develop cilia [61], and it is thought to take up to 1 week for the immature neurons to grow axons to the appropriate target glomerulus in the olfactory bulb of larger animals or humans [85, 92], although this may occur faster in mice, because of the shorter distances [62].

Taken together, the animal studies examining neuro-invasion via the olfactory nerve and olfactory bulb are inconclusive and rather point to alternative routes. Alternative mechanisms of transfer of SARS-CoV-2 from the nose to the brain include the crossing of the blood–brain-barrier after uptake in leukocytes [5, 14, 77, 108], or entering through the endothelial cells of blood vessels [16, 75, 108], reaching CSF-containing spaces associated with the olfactory nerve [14, 19], or by infecting peripheral processes of nervus terminalis neurons that innervate Bowman’s glands, have free nerve endings in the olfactory epithelium, and innervate blood vessels below the olfactory epithelium [9, 19, 55].

## Consequences of brain infection: current controversy

What are the consequences of brain infection with SARS-CoV-2? It is now well established that SARS-CoV-2 can be present—albeit rarely—in the brain of human patients [34, 65, 66, 68, 75, 86, 88], although it needs to be kept in mind that there is an inherent bias because only the most severe (fatal) cases are examined (by autopsy [68, 86]). Not only the route of infection is unclear (as discussed above), but also the consequences of brain infection are currently uncertain, and opinions differ drastically. On the one extreme, it has been proposed that neuro-invasion of the brain may be acutely lethal—animals and patients may die as soon as the brainstem becomes infected, possibly due to shut down of respiratory centers [5, 21, 31, 40, 54, 58, 63, 64, 89]. On the other extreme, it has been noted that brain infection may have little consequence, since there does not seem to be any correlation between the severity of the disease and evidence of the virus in the cerebrospinal fluid (CSF) or brain tissues [65, 74, 86, 88]. An intermediate position is that, as with other viral brain infections, there may be “merely” an increased long-term risk of neurodegenerative diseases due to chronic virus-induced inflammation [30, 32, 39]. It is important to keep in mind that the presence of spike protein or viral RNA does not necessarily mean that the virus actually replicates [1, 82]. It is not yet clear whether the virus or some of the cleaved and circulating viral proteins (spike proteins) typically provoke immune reactions and endothelial cell damage [75] or whether the virus can be present in brains without eliciting any inflammatory or immune reaction (e.g., [89]) or other serious effects such as increased cell death [51]. It is yet uncertain to what extent neurological symptoms in COVID-19 patients are due to a direct viral effect on the brain, or whether neurological symptoms may be primarily due to inflammatory processes, vascular insults, circulating cleaved spike proteins, and other effects of systemic infection in COVID-19 [10, 46, 74–76, 78, 79, 86, 88, 108]. Without a doubt, new insights into these controversies will emerge as the pandemic continues.

## Conclusions

We question an olfactory neuron route of SARS-CoV-2 to the brain for multiple reasons:

- There is a wide consensus that the large majority of mature olfactory receptor neurons do not express the obligatory virus entry proteins.
- Many reports of the virus within olfactory receptor neurons neglect the fact that sustentacular cells tightly wrap these neurons, making it possible to observe false positives even when cell-type-specific markers are used.
- The few infected olfactory receptor neurons reported in some studies are mostly immature cells, but they lack axonal projections to transport the virus into the brain.
- The timeline of neuro-invasion in animal models indicates that the virus uses alternative routes rather than neuron-hopping and virus transfer between olfactory neurons.
- Neuro-invasion of SARS-CoV-2 has consistently been described for a non-physiological mouse model (with transgenic expression of human ACE2 via the K18 promoter), but reports of such neuro-invasion are rare in physiological animal models using the endogenous ACE2 promoter.

Taken together, the current evidence from animal models and human tissues supports the notion that the lack of entry protein expression in olfactory neurons creates a formidable barrier that makes it unlikely for SARS-CoV-2 to gain access to the olfactory bulb along the olfactory nerve axons. It should be noted that this does not rule out a pathway from the nose to the brain by other mechanisms: a vascular route [5, 14, 16, 75, 77, 108], a route through CSF spaces [14, 19], and a route along with the nervus terminalis system [9, 19] or the Grueneberg ganglion [12]. The current evidence favors alternative routes from the nose to the brain, at least in the acute phase (first two weeks) of infection. Since the viral load typically reduces rapidly within the first week of infection [94], the brain appears to be protected in the vast majority of cases with SARS-CoV-2 infection. We are concerned that studies advocating an olfactory route for SARS-CoV-2 to infect the brain may unnecessarily alarm a large number of patients suffering from anosmia. The COVID-19 pandemic is intimidating; our critical review of the evidence indicates that, contrary to several attention-grabbing publications, infection of the olfactory epithelium causing loss of smell in COVID-19 is rarely followed by a brain infection.
